# Improving face identification with specialist teams

**DOI:** 10.1186/s41235-018-0114-7

**Published:** 2018-06-27

**Authors:** Tarryn Balsdon, Stephanie Summersby, Richard I. Kemp, David White

**Affiliations:** School of Psychology, UNSW Sydney, Sydney, NSW 2052 Australia

**Keywords:** Face recognition, Unfamiliar face matching, Identity verification, Individual differences, Personnel selection, Super-recognizers

## Abstract

People vary in their ability to identify faces, and this variability is relatively stable across repeated testing. This suggests that recruiting high performers can improve identity verification accuracy in applied settings. Here, we report the first systematic study to evaluate real-world benefits of selecting high performers based on performance in standardized face identification tests. We simulated a recruitment process for a specialist team tasked with detecting fraudulent passport applications. University students (*n* = 114) completed a battery of screening tests followed by a real-world face identification task that is performed routinely when issuing identity documents. Consistent with previous work, individual differences in the real-world task were relatively stable across repeated tests taken 1 week apart (*r* = 0.6), and accuracy scores on screening tests and the real-world task were moderately correlated. Nevertheless, performance gains achieved by selecting groups based on screening tests were surprisingly small, leading to a 7% improvement in accuracy. Statistically aggregating decisions across individuals—using a ‘wisdom of crowds’ approach—led to more substantial gains than selection alone. Finally, controlling for individual accuracy of team members, the performance of a team in one test predicted their performance in a subsequent test, suggesting that a ‘good team’ is not only defined by the individual accuracy of team members. Overall, these results underline the need to use a combination of approaches to improve face identification performance in professional settings.

**Electronic supplementary material:**

The online version of this article (10.1186/s41235-018-0114-7) contains supplementary material, which is available to authorized users.

## Significance statement

Decades of research shows that people are poor at identifying unfamiliar faces. This is problematic for society because police, government, and private organizations are required to process large numbers of face identification decisions. In these settings, it is common to deploy teams of face identification specialists to adjudicate face identification decisions by comparing images of faces and deciding if they show the same person or different people. In recent years, the selection and recruitment of these teams has been informed by scientific research showing that people vary in their ability to recognize and identify faces, and that this ability is relatively stable over time. This has led to the development of tests to benchmark people’s ability on face identification tests. The purpose of our study was to quantify accuracy improvements on a real-world task afforded by selecting and recruiting high performers for specialist roles on the basis of these standardized tests. We found that benefits of selection were surprisingly modest, suggesting that recruitment based solely on existing tests is unlikely to solve the problem of poor face identification accuracy.

## Background

Many important decisions rely on a person’s ability to identify unfamiliar faces. Does this crowd contain a person of interest? Does the traveler match the person pictured in their passport? Does the suspect match the person shown on CCTV? Many years of research have shown that people make large numbers of errors in these types of tasks. For example, when comparing two images of unfamiliar faces and deciding whether they show the same person or different people, university students make errors on approximately 20% of decisions (e.g., Bindemann, Avetisyan, & Rakow, 2012; Bruce, Henderson, Newman, & Burton, 2001; Burton, White, & McNeill, 2010; Megreya, Sandford, & Burton, 2013; O'Toole, An, Dunlop, Natu, & Phillips, 2012; White, Burton, & Kemp, 2016; White, Kemp, Jenkins, Matheson, & Burton, 2014).

In recent years, it has become clear that these group-level error rates disguise a substantial degree of interindividual variation. On standard tests of face identification ability, some people perform at 100% correct, while others perform near chance (Burton et al., 2010; Russell, Duchaine, & Nakayama, 2009; White, Rivolta, Burton, Al-Janabi, & Palermo, 2017). Similar levels of interindividual variation have been observed in groups of trained professionals that perform face identification in their daily work, such as passport officers (White, Kemp, Jenkins, Matheson, & Burton, 2014; Wirth & Carbon, 2017), and forensic facial examiners (Norell et al., 2015; White, Phillips, Hahn, Hill, & O'Toole, 2015). Critically, this interindividual variation is not merely random noise. Rather, it is predicted by accuracy on other face identification tasks (Bobak, Bennetts, Parris, Jansari, & Bate, 2016; Bobak, Dowsett, & Bate, 2016; Burton et al., 2010; Russell et al., 2009), self-reported ability (Shah, Gaule, Sowden, Bird, & Cook, 2015; Shah, Sowden, Gaule, Catmur, & Bird, 2015), and genomic variation (Shakeshaft & Plomin, 2015; Wilmer et al., 2010). This suggests that the underlying differences giving rise to variation in face identification ability are relatively stable over time.

From a practical perspective, the existence of stable individual differences provides a potential solution to poor levels of accuracy on everyday face identification tasks. Selecting high-performing individuals for professional roles that involve face identification has been advocated in a number of scientific papers on this topic (e.g., Bindemann et al., 2012; Bobak, Bennetts, et al., 2016; Russell et al., 2009; White, Kemp, Jenkins, Matheson, & Burton, 2014). Moreover, a number of professional organizations have now begun to select face identification specialists using standardized tests. A widely publicized example is the ‘super-recognizer unit’ at the London Metropolitan Police Service (Radden Keefe, 2016). Recent tests of individuals in this specialist team show that they outperform university students by an average of around 15% on standard tests of face identification ability (Davis, Lander, Evans, & Jansari, 2016; Robertson, Noyes, Dowsett, Jenkins, & Burton, 2016). A similar group in the Australian Passport Office outperformed students and unselected passport officers by 20% on a test that simulates a task performed in their daily work (White, Dunn, Schmid, & Kemp, 2015, Experiment 2).

While these studies suggest that the selection and recruitment of high performers can provide substantial boosts to accuracy in applied settings, it is not possible to reach this conclusion based on these studies alone. Although there is evidence that these organizations used standard tests of face identification in establishing these groups, there is no scientific record of the processes used to recruit, train, and incentivize these individuals. Many factors could have combined to produce the superior levels of performance observed in these studies; for example, self-selection for the role, training, on-the-job experience, and motivation (see Noyes, Phillips, & O’Toole, 2017). Furthermore, studies of individual differences in face identification typically report the correlation between laboratory-based measures (e.g., Bobak, Bennetts, et al., 2016; Burton et al., 2010), but do not explicitly test the effectiveness of recruitment processes in terms of the accuracy benefits that they produce. As a result, the contribution of recruitment tests towards the high levels of performance seen in specialist teams remains unclear.

In the present study, we aimed to provide the first quantitative guides to accuracy gains that can be expected as a direct result of selecting individuals based on their face identification performance. To do this, we recreated a computerized face identification task performed by professional passport officers to screen passport applications for identity fraud. This real-world task involves participants reviewing lists of images returned by face recognition (FR) software and comparing them with a target image to decide whether any of them match the target. This process is commonly known as ‘FR candidate list review’ in organizations that use this technology for this type of application (see Grother & Ngan, 2014). In a previous study examining performance on this task, we found that experienced passport officers made errors on 50% of decisions (White, Dunn, et al., 2015). Here, we used an updated version of this task to determine whether personnel selection, based on standardized face identification tests, can improve reliability of a real-world passport issuance process.

We present four analyses designed to examine this question. First, we analyze individual differences using a traditional approach by examining patterns of correlation between standard screening measures of face processing ability and the real-world task. Importantly, we verify that these individual differences are stable over time by testing participants in two sessions 1 week apart (see Mollon, Bosten, Peterzell, & Webster, 2017). Second, we apply a variety of selection criteria based on screening test battery performance to examine whether groups of individuals, selected by these criteria, outperform unselected groups on the real-world task. To pre-empt these results, we find that performance on face identification tests were correlated with one another, and were relatively stable across tests performed 1 week apart. However, gains in accuracy conferred by selection are surprisingly modest given the performance of professional specialists reported in previous work. Following on from recent work (White, Burton, Kemp, & Jenkins, 2013; White, Phillips, et al., 2015), in a third analysis we use a ‘wisdom of crowds’ approach and find additive accuracy gains by statistically aggregating decisions of team members. Finally, we examine the stability of this aggregated team performance over repeated tests.

## Methods

The study consisted of two experimental sessions conducted 1 week apart. In the first test session, participants completed a series of tests in the following order: a test designed to simulate a real-world face matching task (FR candidate list review task (FR-Task)), two standardized tests of face identification ability (Glasgow Face Matching Test (GFMT), Cambridge Face Memory Test (CFMT)), and a self-report questionnaire designed to measure their ability to recognize faces (20 item Prosopagnosia Index (PI-20)). Participants then returned 1 week later for a second test session where participants again completed the FR-Task, but this time containing different face images. This enabled us to examine the benefit of selecting participants on the basis of their test scores in session one in terms of their accuracy on the FR-Task 1 week later.

### Participants

One hundred and twenty-three undergraduates from UNSW Sydney participated in the study. Nine participants either did not complete the entire experiment on the first day of testing, or were unavailable for the second day of testing, resulting in a final sample of 114 participants (77 female, mean age = 20.0 years, standard deviation (SD) = 3.46 years).

### FR candidate list review task (FR-Task)

The FR-Task is designed to simulate a task performed in the daily work of passport eligibility officers when using FR software to screen applications for identity fraud. As part of the issuance process at the Australian Passport Office, passport photographs submitted with applications are compared, using FR, against all existing passport photographs in their databases. The FR software then returns a list of images that are the top-ranking matches according to the algorithms’ match-score computation (see White, Dunn, et al., 2015). This list is known as the ‘candidate list’, and passport issuance staff must check this candidate list to ensure that a matching face is not present which would indicate a potentially fraudulent application. In previous work we have shown that average performance of passport officers on this task is very poor and no better than university students (White, Dunn, et al., 2015). In the present study, in order to improve accuracy in this real-world task, we ask which staff should undertake this task, and how best to select them.

The FR-Task was created using a large database of passport images of Australian citizens (*n* = 17,607) who consented for their photograph to be used in research. The database was a random sample of consenting Australian citizens and so was reflective of Australian demographics. We selected two sets of 80 ‘application photographs’ for use in two versions of the FR-Task using item performance data from a previous study (White, Dunn, et al., 2015) to ensure the sets were equated for difficulty. For each application photograph, there was one applicant image and a gallery of four similar looking faces, selected as being the highest ranked images in the passport database according to the face recognition algorithm used by the passport office (Cognitec DBScan 4.1.0 B3). In addition, there was one image of the applicant taken from their previous passport application an average of 9.6 years earlier (SD 2.7 years).

**Fig. 1:**
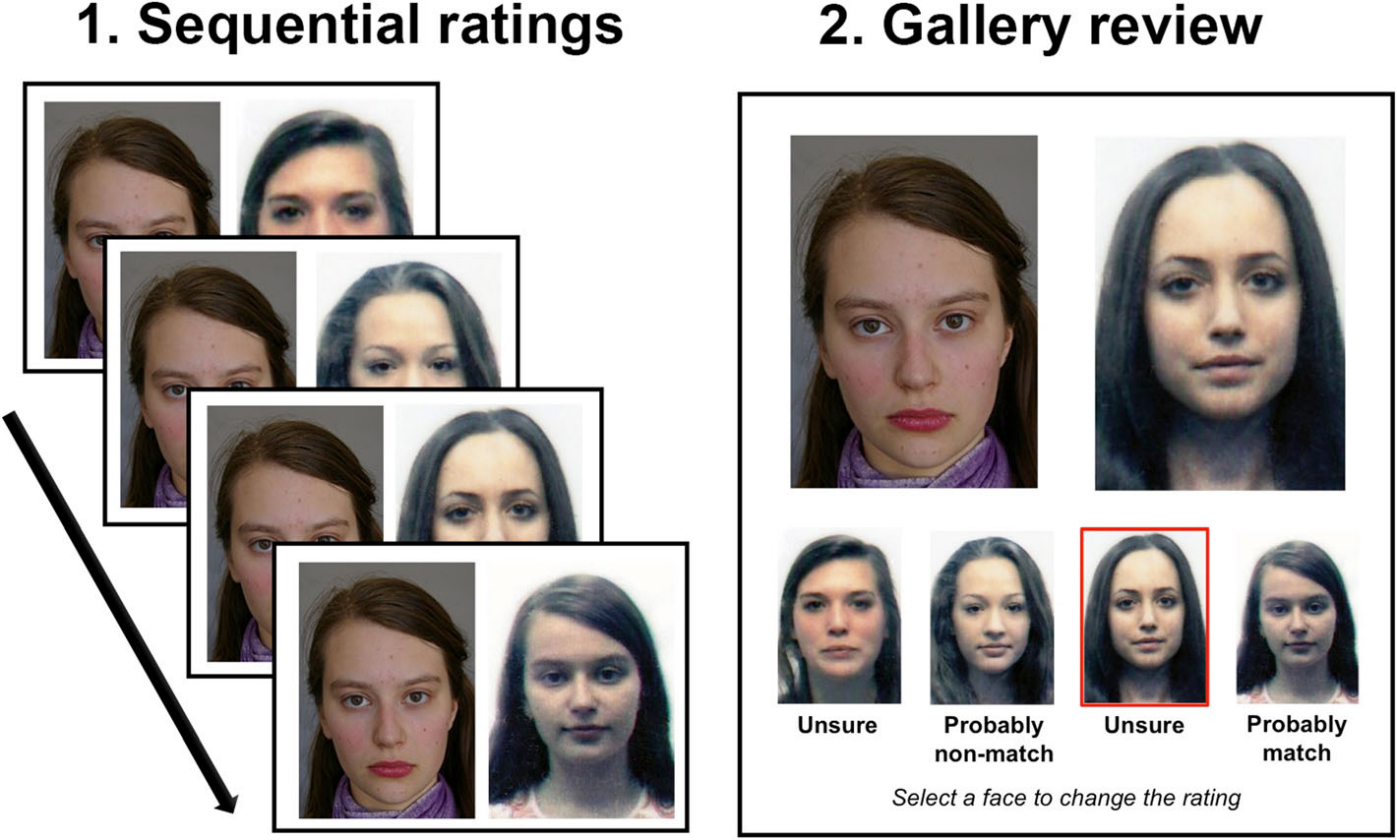
An illustration of the trial design in the Face Recognition Candidate List Review Task (FR-Task). This task simulated the workflow of passport officers using face recognition software to screen for identity fraud in passport applications. On each trial, participants were asked to review four faces that have been flagged by the software as potential matches to the ‘applicant’. Participants made sequential pairwise similarity ratings by comparing the four faces to the applicant (left), before reviewing and confirming these ratings in a gallery review screen (right). The images shown are representative of the images presented to participants, but for privacy reasons we are unable to reproduce the passport images used in the study

The FR-Task was created to simulate the workflow used by passport officers to review the results of FR software in the Australian Passport Office. Each of the 80 trials represented a single passport application, where participants had to compare the applicant photo to four images that had been returned by FR software. Each of these four comparison judgments was made separately, with the applicant image presented on the left of the screen and the ‘candidate’ image on the right. Participants made one of five possible responses to each pair via keyboard: 1) certain nonmatch; 2) probably nonmatch; 3) unsure; 4) probably match; (5) certain match. After they made a response, participants were presented with the next candidate image. Once participants had completed the four comparisons, a review screen was presented with the applicant photo above thumbnail images of the comparison photos. Participants were able to review their decisions by enlarging the thumbnails and then changing their responses before continuing to the next trial. An outline of the trial structure is shown in Fig. 1.

Two versions of the FR-Task were created using different subsets of images, one test for each test session. We attempted to match the difficulty of these tasks using performance data from previous studies. In each version there were 40 target-present and 40 target-absent trials. In target-absent trials, participants compared the applicant to four images of nonmatching candidate identities. In target-present trials, they compared the applicant to three nonmatching candidate identities and a matching target image from the applicant’s previous application. Full-size images in sequential rating screens and comparison images at the top of the gallery review screen (see Fig. 1) were presented at native resolution of 426 × 536 pixels on a 15″ monitor, with resolution 1920 by 1080 (approximately 6 degrees visual angle). Thumbnail images in the review gallery were presented at size 213 × 268 pixels. Each trial contained a different set of stimulus identities, with no repetitions across trials. The order of images in the trial sequence, and the order of trials, were randomized for each participant.^1^

### Glasgow face matching test (GFMT)

The GFMT (Burton et al., 2010) consists of 20 same- and 20 different-identity image pairs. Same-identity pairs show two images of the same person taken under similar lighting conditions, on the same day, but using different digital cameras. Different-identity pairs show two similar looking people. For each image pair, participants respond “same” or “different” identity. The test is self-paced and image pairs remain on the computer monitor until participants make their response. Internal reliability for this test is very high (Cronbach’s alpha = 0.91).

### Cambridge face memory test (CFMT)

In the CFMT (Duchaine & Nakayama, 2006), participants memorize a series of six target face sets and subsequently identify the learned faces from three alternatives on each trial. There are 72 trials in the test, presented in three blocks of increasing difficulty. This test was designed to identify people with impairments in face recognition ability, but has been widely used to examine individual differences across the full-face identification ability spectrum (e.g. Shakeshaft & Plomin, 2015; Wilmer et al., 2010).

### The 20-item prosopagnosia index (PI-20)

The PI-20 (Shah, Gaule, et al., 2015) is a self-report questionnaire designed to assess face recognition ability. Participants were asked to rate the degree to which 20 statements described their face recognition ability (e.g., “*My face recognition ability is worse than most people”*). Participants rated the statements on a five-point Likert scale, with 1 being ‘strongly disagree’ and 5 being ‘strongly agree’. Possible scores range from 20 to 100, with scores from 65 to 74 indicating possible mild developmental prosopagnosia, scores from 75 to 84 indicating possible moderate developmental prosopagnosia, and scores from 85 to 100 indicating possible severe developmental prosopagnosia. As with the CFMT, the primary use of this measure is to identify people with congenital prosopagnosia; however, it has also been successful in identifying higher levels of face matching ability in previous work (Shah, Sowden, et al., 2015).

## Results and analysis

This section is divided into four separate analyses. In Analysis 1, we examined the correlation of the different face identification measures, as is typical in studies of individual differences. In the following three analyses we adopt nontraditional approaches to examining individual differences, with the aim of estimating the potential benefits of selecting high-performing individuals on the basis of their performance in face identification screening tests.

In Analysis 2 we estimate the benefit, in terms of percentage correct improvement, that is conferred by selecting high performers to form specialist teams. We compare the average performance of groups of individuals, selected on the basis of a battery of selection tests, to the grand mean performance on a real-world face identification task. In Analysis 3, we estimate the combined benefit of selecting high performers and then aggregating their independent face identification decisions by response averaging. This is important because it is common in professional organizations for decisions to be made by teams rather than individuals. Finally, in Analysis 4 we examine the variability in performance of face identification teams to begin to understand the factors that contribute to high-performing teams.

### Analysis 1: correlation of face identification ability measures

**Fig. 2:**
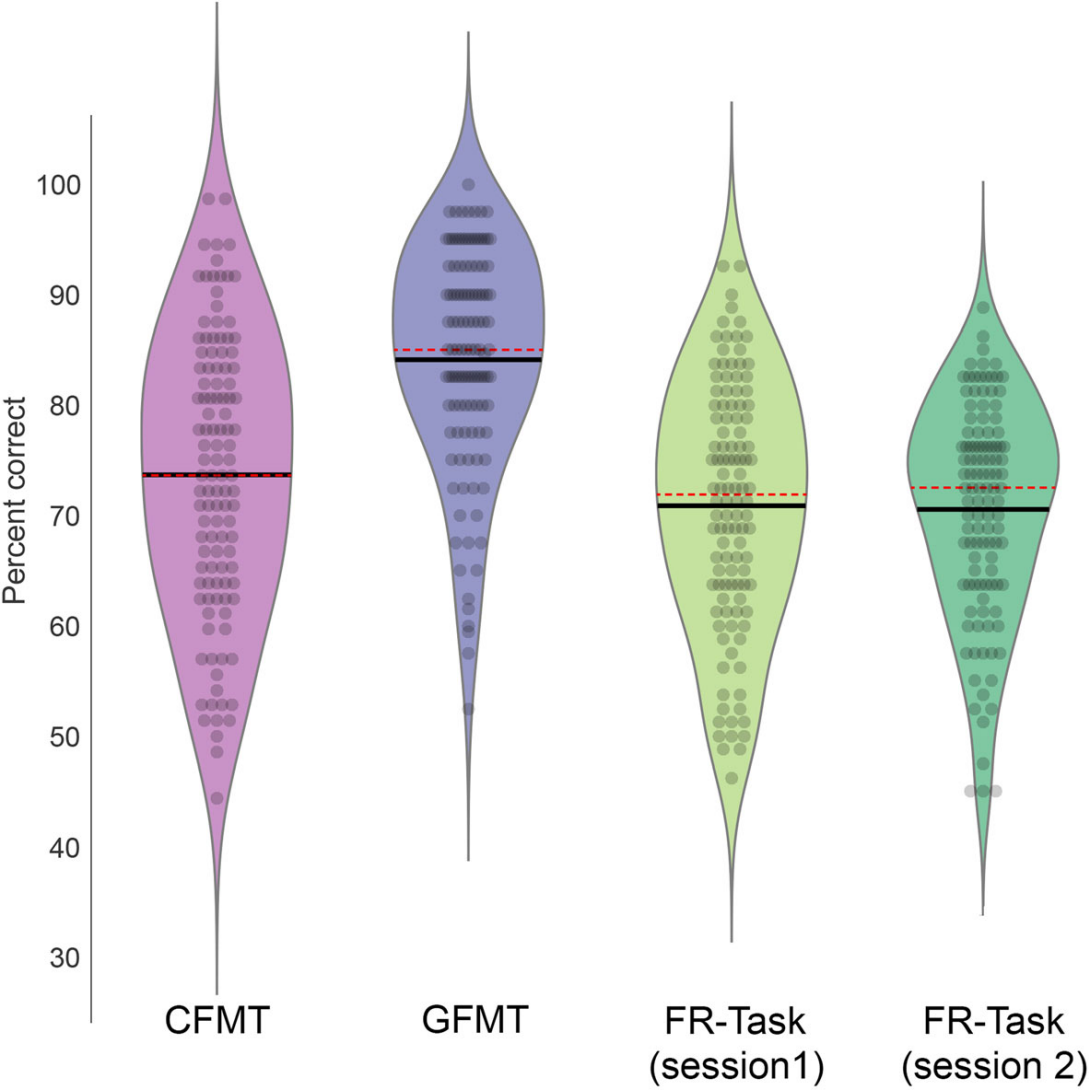
Accuracy distributions for face identification tests. Means are shown in the solid black lines and medians in the red dashed lines. The width of each ‘violin’ represents the expected probability density of that score based on the data and assuming a Gaussian distribution. Individual data points are shown as grey dots. See main text for details. CFMT Cambridge face memory test, FR-Task Face Recognition Candidate List Review Task, GFMT Glasgow face matching test

Accuracy distributions for the four face matching tests are shown in Fig. 2. Consistent with previous work (e.g. Burton et al., 2010; Duchaine & Nakayama, 2006; White, Kemp, Jenkins, & Burton, 2014; White, Kemp, Jenkins, Matheson, & Burton, 2014), we observe large variation in accuracy on all tests that spans the entire range of the performance scale, from chance level (50%) to perfect accuracy (100%). This figure also shows the estimated probability density of performance on each test, represented by the ‘violins’. These density functions are informative, showing that for both the CFMT and GFMT the upper tail of the distribution extends beyond 100%. This is an important result because it suggests the tests are not difficult enough to capture individual differences at the very upper levels of human performance. Although a more challenging version of the CFMT does exist (CFMT+, see Russell et al., 2009), the GFMT short form used in this study is the most difficult version of this test.

Next, we asked whether an individual’s performance on one test of face identification ability predicted their performance on other tests. Correlation between these tests and the PI-20 are shown in Table 1. This table shows moderate correlations between the screening measures (CFMT, GFMT, FR-Task, PI-20). Notably, the correlation between the PI-20 self-report scale and the face tests was roughly equivalent to the correlation between the CFMT and the GFMT, suggesting that this self-report measure captures general face identification ability fairly well (although the correlation is not as strong as in previous studies; see Shah, Sowden, et al., 2015). Importantly, screening tests were only moderately predictive of accuracy on the real-world task of face identification (FR-Task) in both session one and session two. However, correlation between the FR-Task in the first session and the second session was strong, despite different images being used in these two tests. This suggests that while performance on this real-world task is relatively stable across repeated tests, the screening tests used here did not fully capture a person’s ability to perform this task.

**Table 1:** Results of Analysis 1 showing correlation values between face identification ability measures (Spearman’s rho)

	CFMT	GFMT	FR-Task (Session one)	FR-Task (Session two)	PI-20
CFMT	1	0.385	0.325	0.411	−0.344
GFMT	**	1	0.346	0.461	−0.322
FR-Task (1)	**	**	1	0.602	−0.265
FR-Task (2)	**	**	**	1	−0.405
PI-20	**	**	*	**	1

*CFMT* Cambridge face memory test, *FR-Task* Face Recognition Candidate List Review Task, *GFMT* Glasgow face matching test, *PI-20* 20-item prosopagnosia index,

Statistical significance is shown below the diagonal: *p < .01, **p < .001

Previous studies of individual differences in face identification have used correlational measures to test whether performance in one face identification test predicts performance in another (e.g., Burton et al., 2010; Shah, Sowden, et al., 2015). The limitation of this approach is that it does not provide tangible estimates of the gains in accuracy that can be achieved in applied settings by selecting people on the basis of face identification tests. To address this, in the following sections we quantify the extent to which groups of individuals—selected on the basis screening tests—can be expected to outperform unselected groups.

### Analysis 2: benefits of selecting high-performing individuals

In this analysis, we selected groups of individuals based on selection criteria defined by performance on the four face identification measures collected in session one, and then calculated the accuracy of these groups on the FR-Task in session two.

**Fig. 3:**
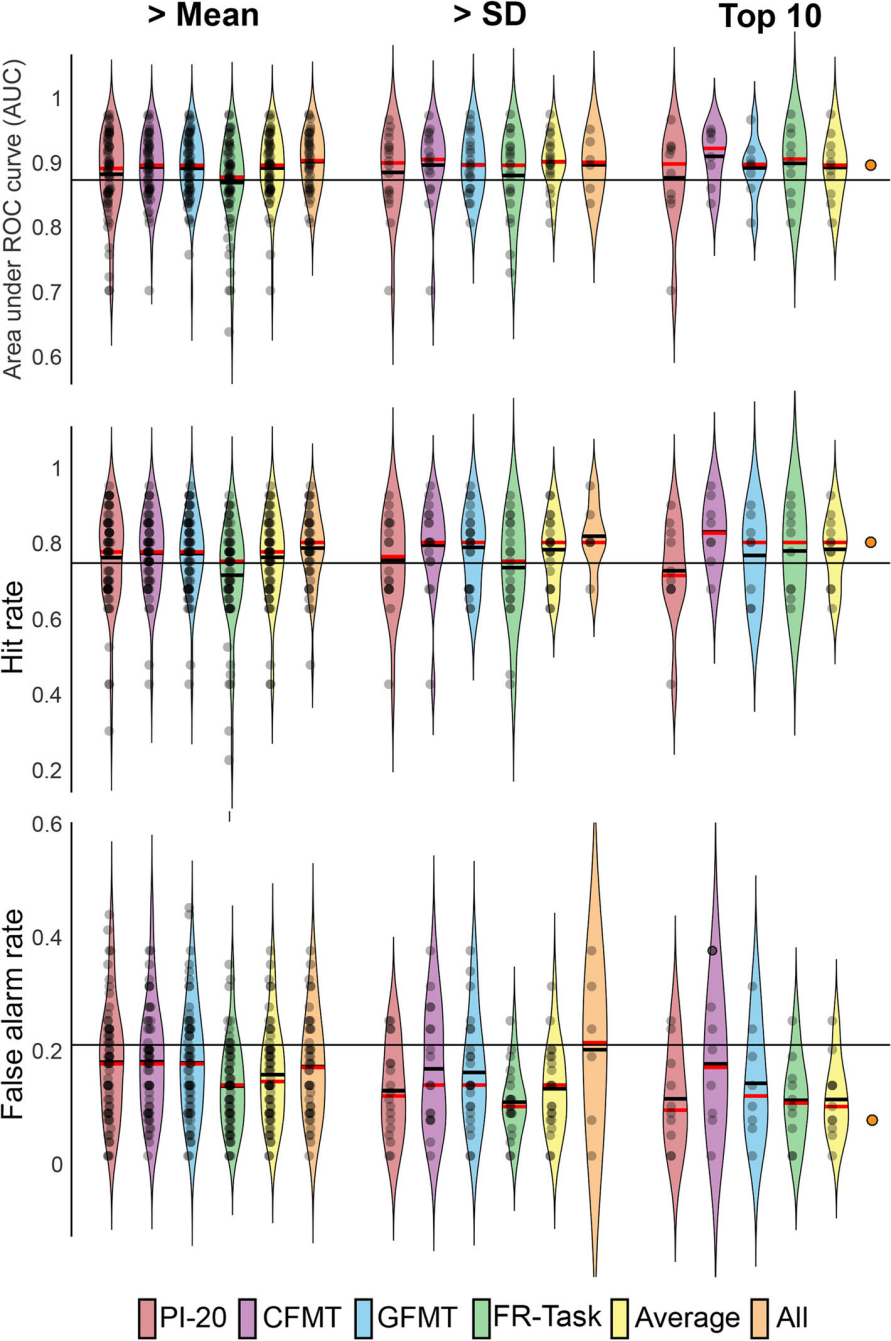
Results of Analysis 2, showing session-two Face Recognition Candidate List Review Task (FR-Task) performance in groups selected using session one screening tests. Continuous black lines show the average of all participants (*n* = 114). Violin plots are presented in three columns pertaining to three selection thresholds (above mean, above one standard deviation (SD), top 10 performers), and show session-two FR Test performance for groups performing above the threshold in each screening measure. Within each violin, black lines represent group mean, red lines are the median, and data points are individual participants. Because only one participant was in the Top 10 performers for face identification accuracy on all screening tests, an orange point shows their performance. CFMT Cambridge face memory test, GFMT Glasgow face matching test, PI-20 20-item prosopagnosia index, ROC receiver operating characteristic

We used three measures to evaluate performance on the FR-Task in session two. Overall accuracy on the FR-Task is represented as area under the receiver operating characteristic (ROC) curve (AUC). We chose this measure instead of percent correct because it provides a measure of accuracy on rating scale responses that is neutral with respect to the decision threshold used. Response scales are often used in applied settings because they enable the decision threshold to be varied, for example in response to varying risk associated with decisions.^2^ AUC scores vary from 0.5, representing chance performance, to 1.0, representing perfect performance. To enable concrete guidance on the benefits of selection, we also express performance in terms of the proportion of Hits and False Alarms. These were calculated using the same method as percent correct in Fig. 3, by taking the highest rating in the gallery, and taking this as a match response if it was ‘unsure’ or greater (see Additional file 1 for a detailed description of each performance measure).

For each measure of face identification ability at session one (CFMT, GFMT, FR-Task, PI-20), we selected individuals whose score exceeded the following cutoffs: i) better that average; ii) at least one standard deviation above the mean; and iii) top 10 performers. We used these same criteria to select individuals on the basis of their average score on the three face identification tests (Average) and also those who consistently exceeded threshold on all three tests (All). These criteria were chosen to represent cutoffs that could be adopted in organizational settings, where recruitment or selection decisions are unlikely to be made on the basis of face identification performance alone, but are instead based on a range of psychometric, interview-based, and resumé-based assessments.

Figure 3 shows session two FR-Task performance scores for each selected group, relative to average performance of all 114 participants. Comparing the violin plots to the continuous black lines provides an indication of the gains in accuracy produced by each of the selection methods. Visual inspection reveals two important aspects of our results. First, for all selection criteria, there are large individual differences in accuracy within selected groups. While the majority of the selected individuals perform better than average on the second test, large proportions perform below average. This is true of all thresholds and all methods for selecting participants, whether based on accuracy on individual face tests (CFMT, GFMT, FR-Task), self-report questionnaire (PI-20), or when selecting on the basis of an aggregate performance score across tests (Average, All).

Second, comparing the overall accuracy of selected groups to the grand mean shows that selection produced surprisingly modest gains. Selecting individuals that had average test scores at least one standard deviation above mean accuracy produced average scores of 78.1% Hits and 13.1% False Alarms in this group, compared with mean performance of all participants of 74.5% Hits and 20.8% False Alarms. Selecting individuals in the Top 10 performers produced very similar levels of performance (78.1% Hits, 11.3% False Alarms). For percent correct, this equates to a 7% improvement when selecting individuals one standard deviation or more above the mean (77.9% vs 70.5%), and a 5% improvement when selecting above the mean (75.3% vs 70.5%).

While benefits of selection appear modest, in professional settings it is possible to rely on the consensus of groups rather than the decisions of individuals. In previous work, we have shown that adopting a ‘wisdom of crowds’ approach by aggregating the face matching decisions of multiple viewers can produce substantial boosts in accuracy (White et al., 2013; White, Phillips, et al., 2015). Thus, in the next section we examine the combined effect of selecting specialist teams and then aggregating their face-matching decisions.

### Analysis 3: benefits of response aggregation using a ‘Wisdom of Crowds’ approach

We performed a ‘wisdom of crowds’ analysis on the session two FR-Task responses of 95 participants from the original sample. This subsample was used because we required every participant to have performed precisely the same versions of the FR-Task at session two for this analysis, and a randomization error meant that 19 participants from the full sample had received slightly different versions at session two.^1^

Two ‘wisdom of crowds’ analyses were carried out, first by sampling teams from the entire group (*n* = 95), and another that sampled from participants who achieved an average score of above one standard deviation on session one face tests (*n* = 17). By comparing performance of selected and unselected teams we aimed to examine the combined effect of selecting high performers and aggregating their responses. For each analysis, we randomly sampled *n* participants and averaged their responses for each image pair presented in the FR-Task separately. This sampling procedure was repeated 1000 times for each value of *n* (2 to 12) and accuracy was computed at each iteration by calculating the group AUC, Hit rate and False Alarm rate. Aggregate accuracy for a given sample size was measured as the average performance across all iterations.

**Fig. 4:**
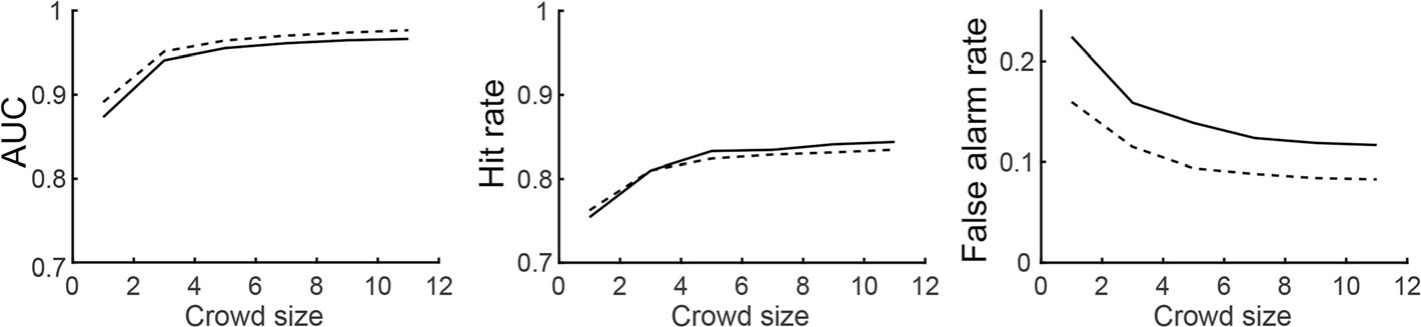
Results of Analysis 3 showing average team performance on FR-Task in session two as a function of the number of individual responses aggregated. The solid line shows teams selected from the whole pool of participants and the dashed line shows teams selected from individuals achieving an average score of above one standard deviation on the session-one face tests. For comparison, crowd size of 1 is included, representing average performance of individuals. Details are provided in the main text. AUC area under the curve

Results of Analysis 3 are shown in Fig. 4. Consistent with previous work (White et al., 2013; White, Phillips, et al., 2015), we found that averaging the scores of small groups produced substantial gains in performance. These gains outweighed benefits of personnel selection when using AUC as a measure. Averaging the responses of just three unselected individuals boosted AUC from .873 to .941, and similar effect sizes were observed for Hit and False Alarm rates. For groups of three individuals performing above one standard deviation on the screening tests, AUC was .963, which is approaching ceiling performance on the test. For both selected and unselected groups, gains in accuracy by response averaging saturated at ‘crowd sizes’ of 8. This is consistent with other recent studies (Jeckeln, Hahn, Noyes, Cavazos, & O'Toole, in press), suggesting there is little value in averaging responses of more than eight individuals.

When using AUC as a measure in Analysis 3, gains afforded by response averaging outweighed benefits of personnel selection. Critically, however, benefits of aggregating decisions were similar for selected and unselected groups, which is consistent with previous studies comparing university students to trained professionals (White, Phillips, et al., 2015). This shows that the benefits of selection and response aggregation are additive, suggesting that these solutions can be used in combination to improve accuracy in real-world tasks.

### Analysis 4: benefits of selecting high-performing teams

Is there such thing as a ‘good team’ of face identifiers? So far, we have shown that selecting teams based on individual performance on screening tests can produce modest gains in group accuracy in a real-world task. Furthermore, aggregating the decision of multiple individuals improves accuracy relative to the average individual performance of the group, providing gains that are additive to those produced by selection alone. This suggests that selecting high performers, and aggregating their decisions, can produce substantial gains in face identification on real-world tasks.

In this final analysis, we ask whether the future accuracy of a team can be predicted by their team performance in previous tests. This analysis was performed on a subset of 54 participants who had completed identical trials at session one and session two. This was because we required every participant to have performed precisely the same versions of the FR-Task in both session one and session two for this analysis.^1^

We randomly sampled 1000 teams of three participants, averaged their responses in session one and two separately, and calculated the accuracy of the team in both sessions from these averaged scores.^3^ As in previous analyses, for the purpose of calculating percent correct, responses of ‘probably match’ and ‘certain match’ were taken as ‘Match’ responses and all other responses as ‘nonmatch’.

**Fig. 5:**
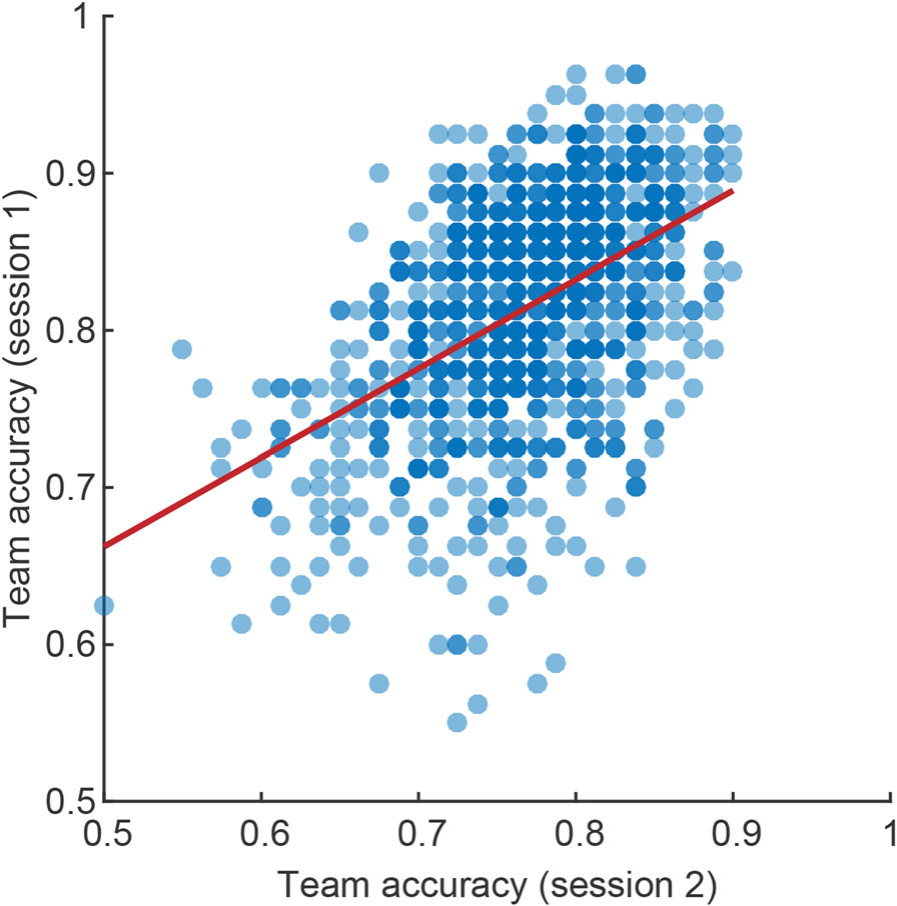
Correlation between FR-Task accuracy of teams in sessions one and two (Analysis 4). Individual data points represent the accuracy achieved by averaging the responses of teams containing three individuals. See text for details

Figure 5 shows the correlation between accuracy of a team in session one and session two. As is clear from visual inspection of this figure, performance of a team in session one is a reliable predictor of team performance in session two. Indeed, the strength of correlation in team performance across repeated tests (*r* = 0.494; *p* < 0.001) is roughly equivalent to the stability seen in individual accuracy reported in Analysis 1.

Naturally, one would expect that individual performance and team performance are related: groups of high-performing individuals will produce superior team performance compared with groups of low-performing individuals. However, it is also possible that some groups will perform better than would be predicted by the accuracy of individual team members, if factors other than individual accuracy predict the accuracy of a team.

One individual difference that may contribute to group performance is cognitive strategy. Theoretical accounts of ‘wisdom of crowd’ effects state that diversity in cognitive strategy and/or information use by individual team members should lead to larger effects (Hong & Page, 2004; Krause, James, Faria, Ruxton, & Krause, 2011; O'Toole, Abdi, Jiang, & Phillips, 2007; c.f. Danileiko & Lee, in press). The proposal is that the more diverse the strategies of team members, the less likely they are to produce correlated errors, resulting in greater benefits of response aggregation. It is therefore possible that certain combinations of individuals produce stronger ‘wisdom of crowd’ effects in face identification tasks. This is important in improving theoretical understanding of ‘wisdom of crowd’ effects in face identification tasks. It is also practically important to know whether—in addition to selecting high-performing individuals for specialist teams—it may also be possible to select combinations of high performers that make good teams.

**Fig. 6:**
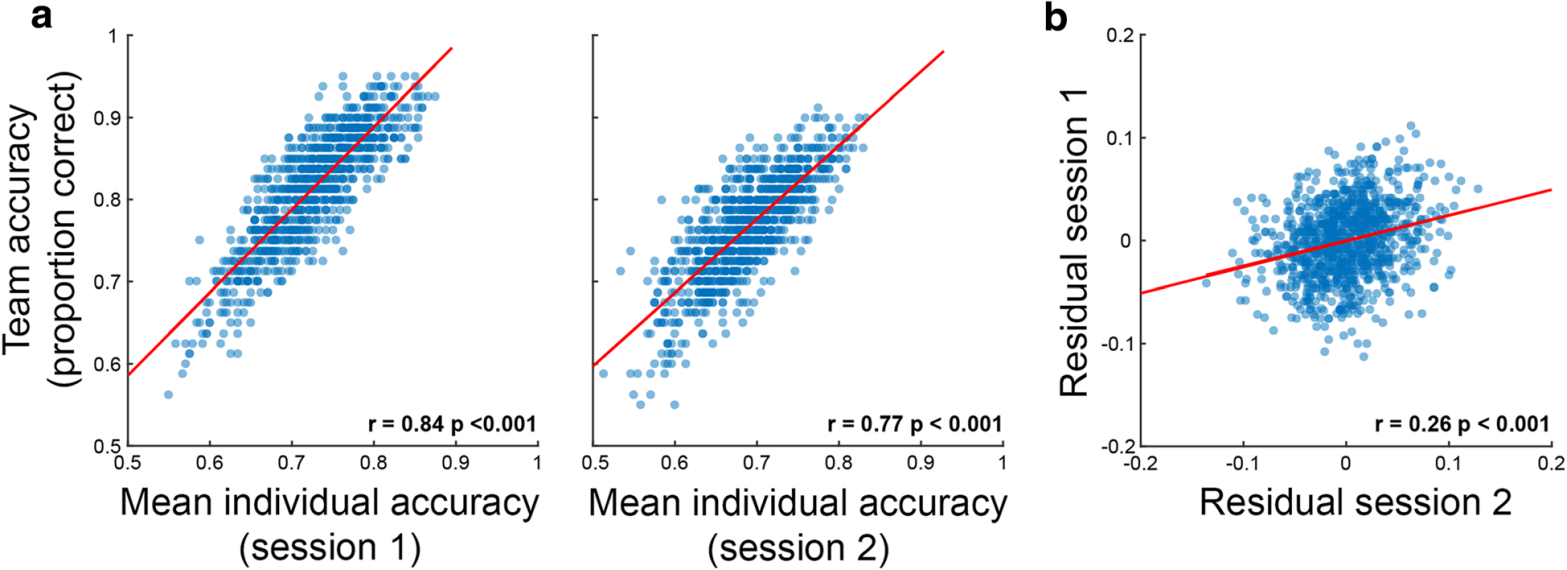
Regression analysis examining the factors underpinning stability in team accuracy across sessions one and two (Analysis 4). **a** Scatterplots showing team performance resulting from response aggregation (*y* axis) as a function of average accuracy of individual team members (*x* axis). Session one data are on the left scatterplot and session two on the right. **b** Scatterplot of residuals from the expected team accuracy based on linear regression in (**a**) for session one as a function of session two. Details of this analysis are provided in the main text

Because accuracy of teams is likely to be highly constrained by the accuracy of individual group members, we first examined this relationship. Figure 6a shows the correlation between the mean individual performance of members in a given team and aggregate team performance—resulting from response averaging—separately for session one and session two. As expected, the correlation between individual accuracy and team accuracy is very strong in both sessions (session one, Spearman’s rho = 0.84, *p* < 0.001; session two, Spearman’s rho = 0.77, *p* < 0.001). These data show that the accuracy of teams is largely determined by the individual accuracy of team members.

However, it is also important to know whether the individual accuracy of team members is the only factor determining team accuracy, or whether other stable characteristics contribute. To address this, we measured the correlation between the residuals in session one and session two scatterplots shown in Fig. 6a by computing the difference of each data point from the regression lines. To the extent that individual accuracy is the only factor predicting a team’s performance, we would expect that residuals would represent random error, and therefore be uncorrelated across test sessions, rather than stable qualities in teams that predict their performance. However, there was a significant correlation between residuals at session one and session two (Fig. 6b, Spearman’s rho = 0.26, *p* < 0.001), suggesting that good teams are not only determined by the individual accuracy of team members.

One individual difference that could be stable across testing and affect the observed benefit of response aggregation is participant response bias. Response bias has been shown to be a stable individual difference on face recognition tasks (Kantner & Lindsay, 2012, 2014). To examine this possibility, we repeated Analysis 4 using the bias-free measure of d-prime. Using this measure, correlation between residuals for session one and session two was nonsignificant (*r* = −0.05; *p* > 0.01; for full analysis see Additional file 1). As we discuss below, this suggests that the stability of team performance is related to differences in the way that individual team members use the response scale.

## Discussion

In this study, we aimed to provide the first estimates of accuracy gains that can be achieved by using tests of face identification ability in recruitment and selection of specialist teams. Consistent with previous studies, we found large individual differences in accuracy, for both standardized laboratory-based tests and in a real-world task. In addition, we observed moderate levels of correlation between the tests and also between test battery scores and subsequent performance in the real-world task performed 1 week later. Rho values between these tests ranged from .3 to .6, which is consistent with those observed in previous studies of individual differences in face identification (e.g., Burton et al., 2010; White et al., 2017).

Despite correlational analysis indicating stability of performance across repeated tests that was in the expected range, we found that the benefit of selecting high performers was quite modest. Specialist groups, selected based on their superior accuracy on a screening test battery, performed on average around 7% better than the overall average performance on the test. At first pass, this appears inconsistent with studies of specialist professional teams, selected using face identification tests, that outperform university students by 10% (Davis et al., 2016), 15% (Robertson et al., 2016) and 20% (White, Dunn, et al., 2015), respectively.

What can explain the disparity between the gains observed in the present study and the superior performance of these professional teams? As noted in the introduction, it is assumed that the professionals were selected on the basis of their performance on standard tests, but there are no known records of such tests. Furthermore, internal selection processes in these organizations were likely to include informal selection based on past performance on case work, and self-selection whereby people choose to pursue a face identification role because they suspect that they have a talent for faces. Given the relatively high correlation between the self-report measure of face identification ability (PI-20) and performance on the real-world face test (FR-Task), it is likely that these informal processes provide useful indicators of future performance on these tasks. This may partly explain why specialist professional groups produce more reliable performance that those individuals selected here on the basis of a single test session.

In a recent review of superior face identification studies, Noyes et al. (2017) proposed that a number of factors may combine to produce high levels of performance. These included natural ability, motivation, formal training, and professional experience. Indeed, recent work has shown that motivation (Moore & Johnston, 2013), training (Towler, White, & Kemp, 2017), and experience collaborating with high performers (Dowsett & Burton, 2015) can all improve accuracy in face identification task. Thus, it appears likely that these factors also contribute to professional accuracy, which may also explain why we observed comparatively modest gains of selection in the current study relative to tests of professional specialist teams.

Nevertheless, the results of this study raise an important challenge for organizations aiming to improve accuracy through personnel selection. If only modest benefits can be expected based on existing recruitment tests, how can these organizations solve the problem of poor face identification? We suggest that the most promising solution is to develop new tests designed to identify high levels of face identification accuracy. Because the benefit of selection is lawfully related to the correlation between test scores and real-world accuracy, improving the correlation between these tests and accuracy in real-world tasks is the most direct route to improving the effectiveness of recruitment-based solutions.

One way to improve this correlation may be to create more challenging tests. For example, Fig. 1 illustrates that the GFMT and CFMT are not challenging enough to capture sufficient resolution at the upper end of the performance distribution, and this is likely to reduce the effectiveness of these tests in selecting the very best face identifiers for specialist roles. We note that a more difficult long-form version of the CFMT is available (CFMT+; Russell et al., 2009; see also Motta-Mena, Elbich, Duchaine, & Scherf, 2017), and future studies should examine whether this test provides greater selection benefits. Another promising avenue is to create tests that model the real-world task that will be performed by recruits. This approach is supported by results of Analysis 1, which show the strongest predictor of performance on the real-word task is performance on this same task, performed 1 week earlier, and containing a different set of faces. Future development of tests that model real-world tasks performed by specialist face identification teams may provide higher levels of correlation than we have observed here.

Notwithstanding this important goal, results of Analysis 3 show that aggregating the responses of multiple viewers can also provide substantial improvements in real-world tasks. Importantly, the benefits of response aggregation were additive to the benefits of recruitment, suggesting that these solutions can be used in combination to optimize accuracy of human face identification decisions. This result is consistent with recent studies of novice participants (White et al., 2013) and professional groups (White, Phillips, et al., 2015) showing that aggregating decisions improves the reliability of face identification decisions.

In Analysis 4, we also found that the performance of teams was stable across two tests separated by 1 week. Importantly, this correlation was not explained by the average individual accuracy of team members alone, suggesting that qualitative differences in the performance of individual team members contributes to team performance. This is an intriguing possibility, and consistent with theoretical accounts of response aggregation benefits which propose that accuracy gains will be greater in teams where different cognitive strategies are used by individual team members (see Hong & Page, 2004; O'Toole et al., 2007). This account is intuitively appealing; the different perceptual processes of individuals will produce different patterns of errors, and so averaging will reduce the impact of these uncorrelated errors on team performance. In machine learning, this principle is well known and accounts for benefits of aggregating decisions of multiple algorithms performing the same task, sometimes referred to as ‘boosting’ (Hastie, Tibshirani, & Friedman, 2001; see Danileiko & Lee, in press) or ‘fusion’ (see O'Toole et al., 2007). This account is especially appealing in light of some evidence suggesting that different people use different perceptual processes to perform face identification tasks, and that these differences can be stable across time (e.g. Richler, Floyd, & Gauthier, 2014).

Although appealing, this account is not supported by our results. Critically, stability of team performance in Analysis 4 was only observed when using overall accuracy as the dependent measure. When we instead used a bias-free measure of accuracy (d-prime; see Additional file 1), the pattern was not observed. We propose that this is an informative difference, suggesting that the stable factor producing ‘good teams’ is related to response bias of individual team members. One possibility is that averaging the responses of teams serves to moderate extreme response biases of individual team members. For example, if one team member has a tendency to respond ‘match’ more than is optimal, and another has the opposite bias, then averaging would serve to better calibrate the responses of teams to the scale.

Previous tests of forensic face identification experts have shown systematic differences in their use of response scales relative to novices (see Hu et al., 2017; Norell et al. 2015), suggesting that response behavior is changed by professional training and experience. Given that aggregation of individual responses appears to be a useful approach to improving face identification in professional settings (see Hu et al., 2017; White et al., 2013; White, Phillips, et al., 2015), it will be important in future work to develop an understanding of the relationship between individual response behavior and team accuracy. It is also theoretically important to determine whether, in visual comparison tasks more generally, ‘wisdom of crowd’ effects arise from diversity in the perceptual or decisional processing of individual team members.

## Conclusions

Overall, our results underscore the need to use a combination of approaches to improve performance in applied settings. In recent years, cognitive research has shown that training (Towler et al., 2017; White, Kemp, Jenkins, & Burton, 2014), teamwork (Dowsett & Burton, 2015; Jeckeln et al., in press), response aggregation (Jeckeln et al., in press; White et al., 2013; White, Dunn, et al., 2015; White, Phillips, et al., 2015), and familiarization (Dowsett, Sandford, & Burton, 2016; Murphy, Ipser, Gaigg, & Cook, 2015; Ritchie & Burton, 2017; White, Burton, Jenkins, & Kemp, 2014) can all improve accuracy of unfamiliar face identification. Therefore, selection should not be viewed in isolation from these other approaches. To address the problem of poor human performance in important face identification tasks, professional organizations can draw on a variety of evidence-based solutions.

## Additional file

Additional file 1: Supplementary Methods and Analysis. (DOCX 2931 kb)

## Footnotes

1 In the FR-Task we randomized whether a particular applicant identity was presented as a target-present (where previous application photo was presented) or target-absent trial (previous application photo not presented). This randomization was performed for some participants but not others (60 in session one and 19 in session two). We had not intended to randomize this and so we turned randomization off when the coding error was detected. The study was designed so that participants would each be shown precisely the same versions of the task, as this was critical for response aggregation in Analysis 3 and 4. This led to data from 95 participants being analysed in Analysis 3 (those who received no randomization in session two), and 54 for Analysis 4 (no randomization in either session). Comparing data from participants who did and did not receive this randomization confirmed that it did not affect the results of Analysis 2 (see Additional file 1: Figure S1).

2 It was not possible to use this measure on tests other than the FR-Task, and so for the purposes of the correlations in Analysis 1 we computed accuracy as percent correct. For further details of how performance measures were calculated please refer to Additional file 1.

3 It was necessary to use percent correct rather than AUC for this analysis, as ceiling effects were present in AUC scores of high-performing groups that caused a nonlinear relationship between team accuracy and individual accuracy (see Additional file 1). We also repeated the entire analysis for team size of 6 and found the same pattern of results.
