# Supplementary material for: Improving face identification with specialist teams
Source: Cogn Res Princ Implic. 2018 Jun 27;3:25. doi: 10.1186/s41235-018-0114-7 (PMC6021458; doi:10.1186/s41235-018-0114-7)
Supplement: Supplementary file 1 — Supplementary Methods and Analysis. (DOCX 2931 kb) [file 41235_2018_114_MOESM1_ESM.docx]

**Improving face identification with specialist teams**

**Supplementary Methods and Analysis**

Tarryn Balsdon, Stephanie Summersby, Richard Kemp & David White*

*^*^Corresponding author: david.white@unsw.edu.au*

**Performance measures for the FR-Task**

***Hit Rate.*** Number of hits was calculated as the number of target present trials in which participant’s highest rating was given to the matching face, where the highest rating was ‘Probably Match’ or ‘Certain Match’. To calculate hit rate, we divided by the number of hits by the number of target present trials (40).

***False alarm rate.*** Number of false alarms was calculated as the number of times a participant’s highest rating in a gallery was given to a non-matching face, where the highest rating was ‘Probably Match’ or ‘Certain Match’. Because participants could make false alarm responses on both target present and target absent trials, false alarm rate was calculated as the number of false alarms divided by the total number of galleries presented (80). Further, a participant could score both a false alarm and a hit on a single trial if they made more than one match response, and one of these images was of the target face. In the rare cases that this occurred, both a hit and a false alarm were counted.

***Percent correct.*** Percent correct was calculated as number of Hits, plus the number of target absent trials in which the participant did not make a match response, divided by the total number of trials (80).

***Area under the ROC curve (AUC).*** AUC is a bias free measure of sensitivity to discriminating target stimuli from distractors. The proportion of each rating response when the participant is presented with the target stimulus is plotted against the corresponding proportions when presented with distractor stimuli, forming a polygon that ranges in area from 0.5 to 1. An area of 0.5 suggests that the participant did not differentiate between target stimuli and distractor stimuli in their ratings (they were equally likely to give a high rating to a distractor as they were to a target). An area of 1 suggests that the participant perfectly discriminated between targets and distractors with their ratings (they gave high ratings only to targets and low ratings only to distractors).

***D-prime.*** Like AUC, d-prime (d’) is an index of sensitivity to discriminating target stimuli from distractors. Based in Signal Detection Theory (SDT), d’ assumes that the evidence for target presence emerges from the stimulus and additive Gaussian noise, and is represented on an internal continuum and compared to some criterion for making a decision. The model assumes that when the target is absent, the Gaussian noise can push the evidence past the criterion, causing a false alarm. Simple calculations of d’ take the difference in the normalized hit rate and false alarm rate, assuming equal variance in the Gaussian noise across target present and target absent trials. This assumption is rarely valid, so for pairwise responses we calculated d_a,_ by fitting a receiver operating characteristic (ROC) curve to participant’s rating responses (plotted as for AUC above) using RScorePlus (version 5.6.1, Harvey, 2001; algorithm from Dorfman and Alf, 1969). Chance performance yields a d’ of 0, but there is no upper limit to d’.

**Supplementary Analysis**

Because of a coding error that randomized the allocation of target present and absent trials to stimulus identities, the first 60 participants each received a slightly different version of the FR-Task (see Endnote A, main paper). This may have affected the observed stability of individual differences across session one and session two performance on the FR-Task. To address this, we repeated Analysis 2 with only the 54 participants who completed exactly the same version of the FR-Task. This analysis is shown in Figure S1 and shows that the benefits of selection in this sample did not exceed those that were reported in Analysis 2.


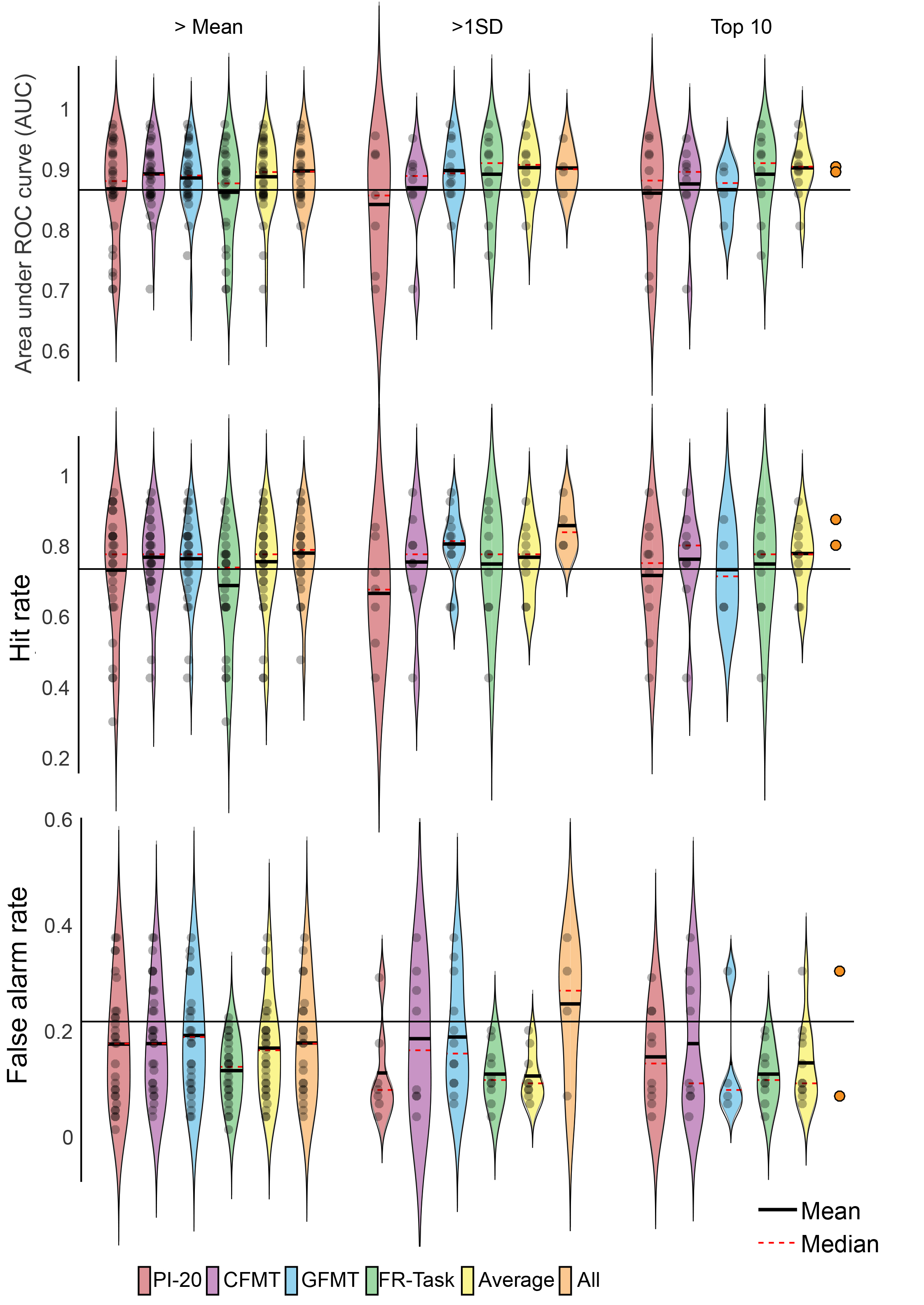


***Figure S1. Results of Analysis 1 for the subset of 54 participants that completed identical versions of the FR-task in sessions one and two.*** *Results are* *consistent with those presented in the main paper, based on the full sample of 114 participants. Details of this analysis are provided in the main text of the paper.*

Analysis 4 in the main paper examined whether team performance was stable across repeated testing. An initial analysis of AUC scores revealed ceiling effects for high performing groups (see Figure S2), and so we performed this analysis using percent correct as a measure of performance.

**
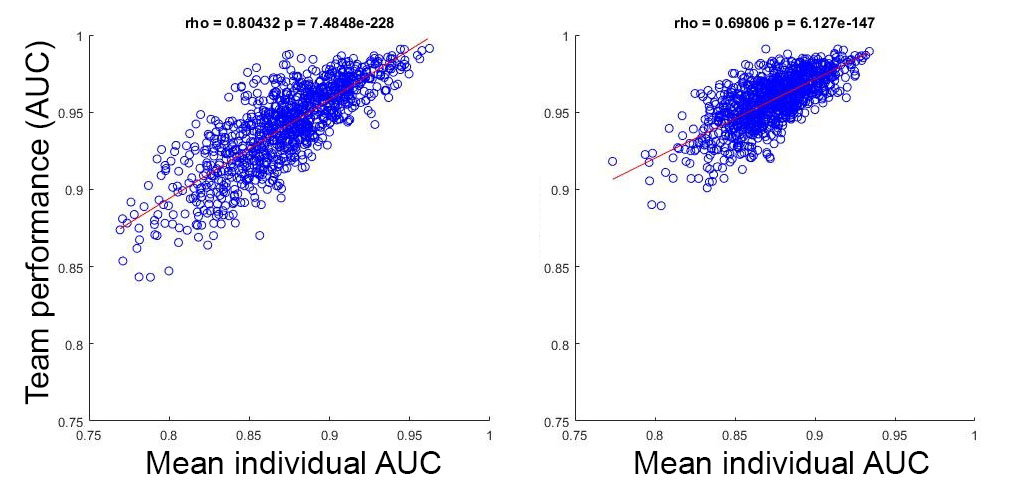
**

***Figure S2. FR-Task AUC scores for teams of three plotted as a function of the mean individual accuracy for session one (left) and session two (right).*** *Ceiling effects were apparent for the highest performing teams, causing unequal variance, and so percent correct and d-prime were used for this analysis.*

We chose percent correct and AUC as the primary measures of performance as they provided an indication of the magnitude of errors that can be expected in applied settings. However, the use of percent correct in Analysis 4 meant that response bias of individual team members could have affected the observed stability in team performance. Therefore, we repeated this analysis with a bias-free measure of accuracy, d-prime. Results of this analysis are shown in Figure S3 (for details of d-prime measure see the previous section).

Figure S3B shows the correlation of residuals from plots shown in Figure S3A. As in Analysis 4, the degree of correlation between residuals represents the variance in team performance – independent of individual team members’ accuracy – that is stable across tests. Residuals were not correlated significantly when repeating this analysis with d-prime (r = -0.05; p > 0.1), with residuals therefore representing random variations in team performance across the sessions. This suggests that the stability of team performance reported in Analysis 4 is related to response bias. For further discussion of this result please see the Discussion.


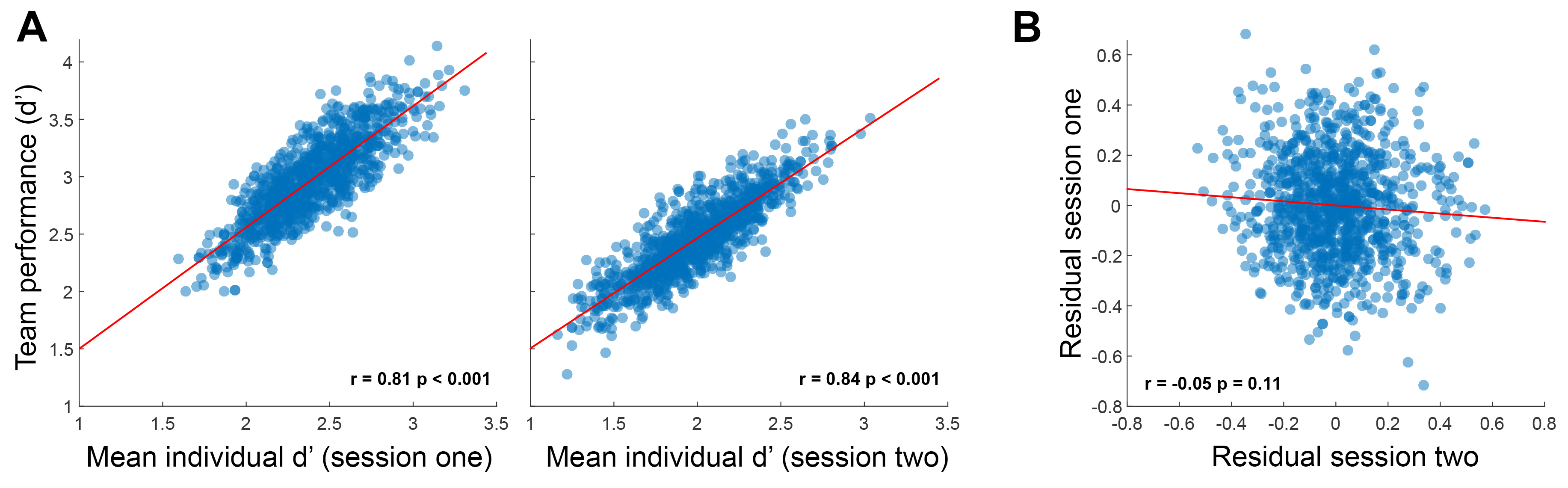


***Figure S3. Regression analysis examining the stability of team accuracy across sessions one and two using d-prime as performance measure.*** *(A) Scatterplots showing team performance resulting from response aggregation on the y-axis, as a function of average accuracy of individual team members on the x-axis. Session one data are on the left scatterplot and session two on the right. (B) Scatterplot of residuals from the expected team performance based on linear regression in (A) for session one as a function of session two accuracy. See text for details.*

**References**

Dorfman, D. D., & Alf, E. (1969). Maximum-likelihood estimation of parameters of signal-detection theory and determination of confidence intervals—Rating-method data. *Journal of Mathematical Psychology, 6*(3), 487-496.

Harvey, L. O., Jr. Parameter estimation of signal detection models: RSCORE PLUS user’s manual [Computer software manual]. Boulder, CO: Author. 2001.
